# *Bacillus subtilis M6* improves intestinal barrier, antioxidant capacity and gut microbial composition in AA broiler

**DOI:** 10.3389/fnut.2022.965310

**Published:** 2022-08-17

**Authors:** Linbao Ji, Lian Zhang, Hu Liu, Jiakun Shen, Yu Zhang, Lin Lu, Xiujun Zhang, Xi Ma

**Affiliations:** ^1^State Key Laboratory of Animal Nutrition, College of Animal Science and Technology, China Agricultural University, Beijing, China; ^2^College of Animal Science and Technology, Beijing University of Agriculture, Beijing, China; ^3^School of Public Health, North China University of Science and Technology, Qinhuangdao, China

**Keywords:** *Bacillus subtilis*, antibacterial activity, intestinal barrier, antioxidant, gut microbiota

## Abstract

*Bacillus subtilis* can secret a variety of substances to improve human and animal gut health *via* inhibiting the proliferation of pathogenic bacteria. In this study, a fast-growing and stress-resistant strain of *Bacillus subtilis M6* (*B. subtilis M6*) were isolated, which showed a strong antibacterial activity to *E. coli* K88, *S. typhimurium* ATCC14028, and *S. aureus* ATCC25923 *in vitro*. *In vivo* studies showed that *B. subtilis M6* can significantly improve the average daily gain (ADG) using an AA broiler model. Dietary *B. subtilis M6* improved the intestinal morphology. The villus height of jejunum and ileum were significantly increased. The concentration of malondialdehyde (MDA) in the ileal mucosa was significantly reduced in *B. subtilis M6* treatment group, which suggested the oxidative stress of the ileum was significantly relieved. Though the β diversity of treatments was not significantly, *B. subtilis M6* improved the composition of intestinal microbes, especially at the level of caecum genus, the dominant genus was changed from *Ruminococcus* to *Akkermansia*, which indicated the change of intestinal carbohydrate nutrition. In conclusion, these data indicate that the *B. subtilis* M6 shows a probiotic potential to improve intestinal health *via* altering gut microbiota.

## Introduction

Probiotics are a kind of living microorganisms that can be colonized in the digestive tracts of human and animals, and further improve their health. The diversity and richness of probiotics is very important in regulating human health (1). Numerous studies have confirmed that probiotics can have a preventive or therapeutic effect on many diseases. For example, a multi-strain probiotic or a mono-strain supplement can improve symptoms in irritable bowel syndrome (IBD) patients after up to 8 weeks of use (2). Gut microbiota plays a distal immune modulation role in relieving respiratory disease by the gut-lung axis. In addition, it has been reported that Alzheimer's disease (AD) may be associated with a dysbiosis of microbes in the intestine (3). These positive effects can be achieved by probiotics because they are successfully colonized in the gastrointestinal tract of the host (4).

With the in-depth research related to probiotics, people began to pay attention to safety issues. Bimodal probiotic strains of the *Bacillus* genus, which are typically found in soil, water, and various non-dairy fermented foods, as well as in human and animal guts, constitute the microbiota of the human environment (5). Furthermore, along with expanded usage of probiotics and improved tracking methods to specific strains, the reports of infections and other adverse incidents were increased (6). Carrying genes for various toxins or antibiotic resistance, and various strains can pose a substantial health risk (5).

Antibiotics are normally used to remove or prevent bacterial colonization in the human body without targeting specific types of bacteria. As a result, broad-spectrum antibiotics can greatly affect the composition of the gut microbiota, reduce its biodiversity, and delay bacterial colonization for a long period after administration (7). More than 50 years ago, antibiotics have been widely used in commercial poultry production as feed additives because they can improve feed efficiency and animal growth by regulating intestinal microbiota and preventing diseases by improving immunity (8). However, the antibiotics deposited in food will eventually act on the human body and pose a threat to human health. The wide application of antibiotics has brought more and more challenges, such as environmental pollution and the development of bacterial antibiotic resistance. Therefore, growth-promoting antibiotics have been banned as feed additives in Europe since 2006, the United States since 2014, and China since 2020 (9).

In the present study, we used AA broilers as a model to test whether *B. subtilis* M6 supplementation would have a positive effect on the host and to lay the groundwork for its study in humans.

## Materials and methods

### Morphological and biochemical identification of *B. subtilis M6*

#### Observation of colony morphology

*Bacillus subtilis M6* was isolated from the soils around the Laboratory of Swine Metabolism of China Agricultural University (Beijing, China). Briefly, the bacterial solution was diluted for an appropriate multiple, then applied to LB solid medium and incubated at 37°C in an incubator for 16 h. The sizes, color, shape, edge integrity, and transparency of the colonies were recorded.

#### Observation of bacterial morphology

Firstly, a drop of distilled water was applied onto the slide. Secondly, a single colony were selected and fully mixed in the distilled water. The slide was hold and quickly moved through the alcohol lamp several times to fix. Thirdly, the slide was stained with crystal violet for 30 s, washed with distilled water 3 times, and then checked under the microscope.

### Growth characteristics and tolerance test

#### Growth characteristics

Select a single colony of streak plate method cultured *B. subtilis* and culture it in LB medium with shaking at 37°C, then inoculate the fresh bacterial suspension in LB liquid medium at the proportion of 1% and culture it in a 37°C incubator with shaking. Determine the pH value and the number of viable bacteria at OD 600nm every 2 h. The plate counting method is used to calculate the number of viable bacteria.

#### Acid resistance

The low pH tolerance was evaluated, 1% activated strains were incubated with LB medium with different pH values (2.0, 3.0, 4.0, 5.0). The mixture was incubated at 37°C for 1, 2, 3, and 4 h, and the number of viable bacteria was calculated by the plate colony counting method. Taking the viable count of 1% of the activated strain in sterile LB liquid medium without any treatment as a control, calculated the survival rate of *B. subtilis M6* in different pH conditions levels.

#### Bile salt tolerance

Activated *B. subtilis M6* suspension was added at 1% to LB medium containing various concentrations of porcine bile salts (0.0, 0.1, 0.2, and 0.3%). The mixture was put under the condition of 37°C for 1, 2, 3, and 4 h, respectively. Each treatment was repeated 3 times. The number of viable bacteria tolerated for different times was calculated by the plate counting method. Taking the viable count of 1% of the activated strain in sterile LB liquid medium without any treatment as a control, calculated the survival rate of *B. subtilis M6* in different bile salt conditions levels.

#### High-temperature resistance

After the activation of *B. subtilis M6*, the fresh bacterial suspension was placed into the water bath pot at different temperatures (70, 80, 90, and 100°C) for 0, 3, 5, 10, and 15 min respectively. Each treatment was repeated three times. The number of viable bacteria tolerated at different times was calculated by the plate counting method. Finally, the number of 0 min was used as a control. The survival rate of the strain under different high-temperature conditions for a certain time was calculated.

### Antibiotic sensitivity assay

Antibiotic sensitivity was determined by drug-sensitive paper tablets (Hangzhou microbial reagent CO, LTD, China). Select 1~2 common antibiotics from each class as a representative to comprehensively reflect the drug sensitivity of *B. subtilis M6*. Then 1% *B. subtilis M6* was mixed in melted LB solid medium till it solidified and dried for 3~5 min. The tablets were homogenized spread on the plate and gently compacted, with a spacing of no <24 mm, sheets at the center from the edge of the plate 15 mm, which prevents the crossing between each transparent circle. Every tablet was repeated in triplicate. The circle diameter that <15 mm was resistant and 16~20 mm for medium sensitive, the bacteriosphere diameter of >20 mm is sensitive.

### Bacteriostasis test

After the indicator bacteria were activated three times, a single colony was inoculated into the LB medium and cultured in a shaking table at 37°C, 220 rpm for 1 h to reach 1 × 10^8^ CFU/ml. Then 1% of the inoculation amount was mixed in LB solid medium and perforated with an Oxford cup. *Bacillus* was inoculated in LB liquid medium at a ratio of 1% and cultured at 37°C for 16 h. The cultured bacillus was centrifuged with 3,000 × g for 8 min at 4°C, the supernatant and suspension were collected respectively, and the bacteria were resuspended with sterile normal saline. 150 μl normal saline, bacterial fermentation broth, supernatant, and bacteria were added to the holes made with Oxford cup in advance, and they were cultured in incubator for 12 h at 37°C. Then the diameter of the bacteriostatic circle was measured.

### Animal experimental and sample collection

All the procedures of this experiment were approved by the animal protection and utilization organization committee of China Agricultural University (AW90602202-1-1). 324 1-day-old AA broilers were randomly divided into three treatment groups: blank control group (CON, basic diet), positive control group (CON+, basic diet + 200 ppm aureomycin), and treatment group [TRT, basic diet + 0.5 g/kg *B. subtilis M6* (1 × 10^9^ CFU/g)]. Each treatment had 6 replicates and 18 chickens per replicate. The experimental period was 42 days. The survival status of AA broilers was observed and recorded. The weekly weight change and feed intake change of AA broilers were recorded, and the average daily gain, average daily feed intake, and feed weight ratio was calculated according to the daily weight change and feed intake.

After 42 days of feeding, the jejunum, ileum, and cecum were collected. About 1 cm of the intestinal segment was placed in paraformaldehyde as fixed samples for subsequent tissue sectioning experiments. The chyme of each intestinal segment was collected in a 2 ml cryopreservation tube and snap froze in liquid nitrogen. The samples were stored at −80°C for subsequent 16S rDNA sequencing. The jejunum and ileum segments were cut, rinsed in normal saline, and unfolded horizontally. The intestinal mucosa was scraped with a slide and put into a 2 mL centrifuge tube. These samples were froze in liquid nitrogen and stored at −80°C.

### Morphological analysis of intestinal mucosa

The morphology of intestine histological sections stained with hematoxylin and eosin (H&E) were observed by invert microscope (Olympus BX51, Japan) and the length and depth of intestinal villus were measured by the image analysis program. Moreover, the ratio of villus length to crypt depth was also calculated.

### Microbiota profiling

Microbial genomic DNA from colon chyme was extracted by commercial Bacteria Genomic DNA Kits (Solarbio, Beijing, China). Genomic DNA was amplified using specific primers with the barcode locked in the 16S V3-V4 region. Paired-end sequencings were read through the Illumina MiSeq platform. An OTU table was obtained from the Mothur Bayesian classifier. The original sequencing data were filtered and processed to get the valid data, in which the DADA2 method recommended by QIIME2 was applied to de-noise, merge, and de-chimera. Taxonomy classification and taxonomic analyses referring to the Silver database were performed based on the OUT table. Then, the abundance and diversity index of OTU were analyzed. Beta diversity analysis were conducted to get principal coordinates and visualize. Shannon and Chaol indexes are used to evaluate the complexity of species diversity.

### Antioxidant indexes of jejunum and ileum

Jejunal and ileal mucosal were weight about 0.1 g and added 900 ul sterile normal saline to form 10% tissue homogenate. The homogenate was centrifuged at 3,000 × g and 4°C to obtain the supernatant. The supernatant was recorded the volume, measured and corrected the protein concentration according to the instructions of the kit. Furthermore, MDA (malondialdehyde), SOD (superoxide dismutase), and T-AOC (total antioxidant capacity) in the intestine were determined according to the kit of Nanjing Jiancheng Company.

### Statistical analysis

Statistical analysis was carried out using SAS version 9.1 (SAS Institute Inc, Cary, NC, USA). ANOVA followed by Tukey's multiple range tests was performed to assess statistical significance. The results were shown as mean ± SEM. *P* < 0.05 was considered significant, and *P* < 0.01 was strongly significant. All raw sequence datasets have been uploaded to the NCBI Sequence Read Archive (SRA) with the accession number PRJNA847429.

## Results

### Colony morphology and prebiotic potential of *B. subtilis M6*

#### Identification of *B. subtilis M6*

**Figure 1:**
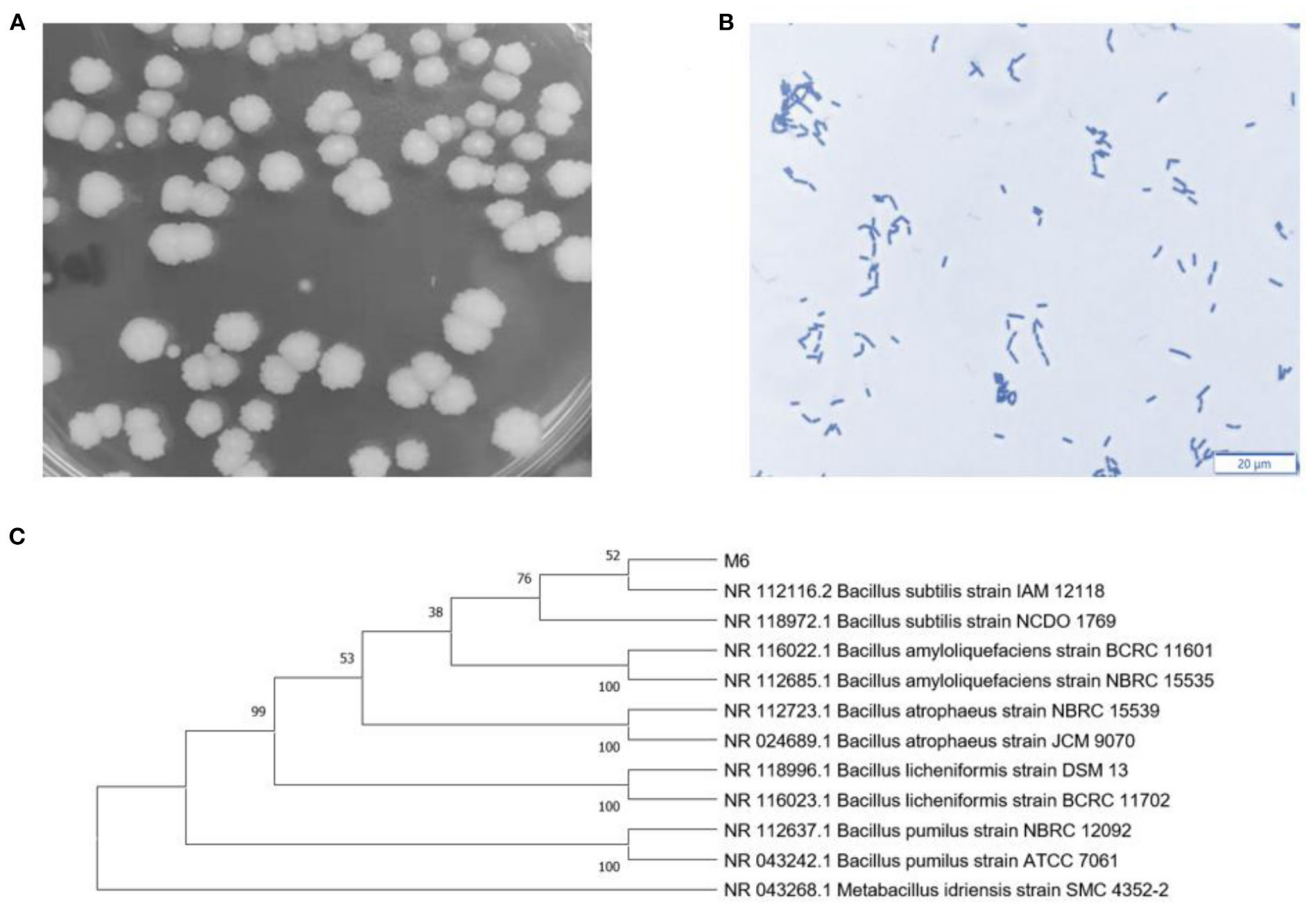
Identification of *B. subtilis M6*. **(A)** Colon morphology of *B. subtilis M6*. **(B)** Gram staining of *B. subtilis M6*, bar = 20 μm. **(C)** The phylogenetic tree of *B. subtilis M6*.

The colony morphology is Milky white and round, with dry surface folds, opacity, and irregular edges (Figure 1A). The strain was stained with crystal violet and observed under an oil microscope (100 ×). The bacteria are rod-shaped, with a size of about 2~5 μm (Figure 1B). Sequencing results 16S rDNA sequence were submitted to NCBI database for BLAST detection and were analyzed consanguinity *via* phylogenetic tree (Figure 1C). Therefore, the strains were named *B. subtilis M6*.

#### Growth characteristics of *B. subtilis M6*

**Figure 2:**
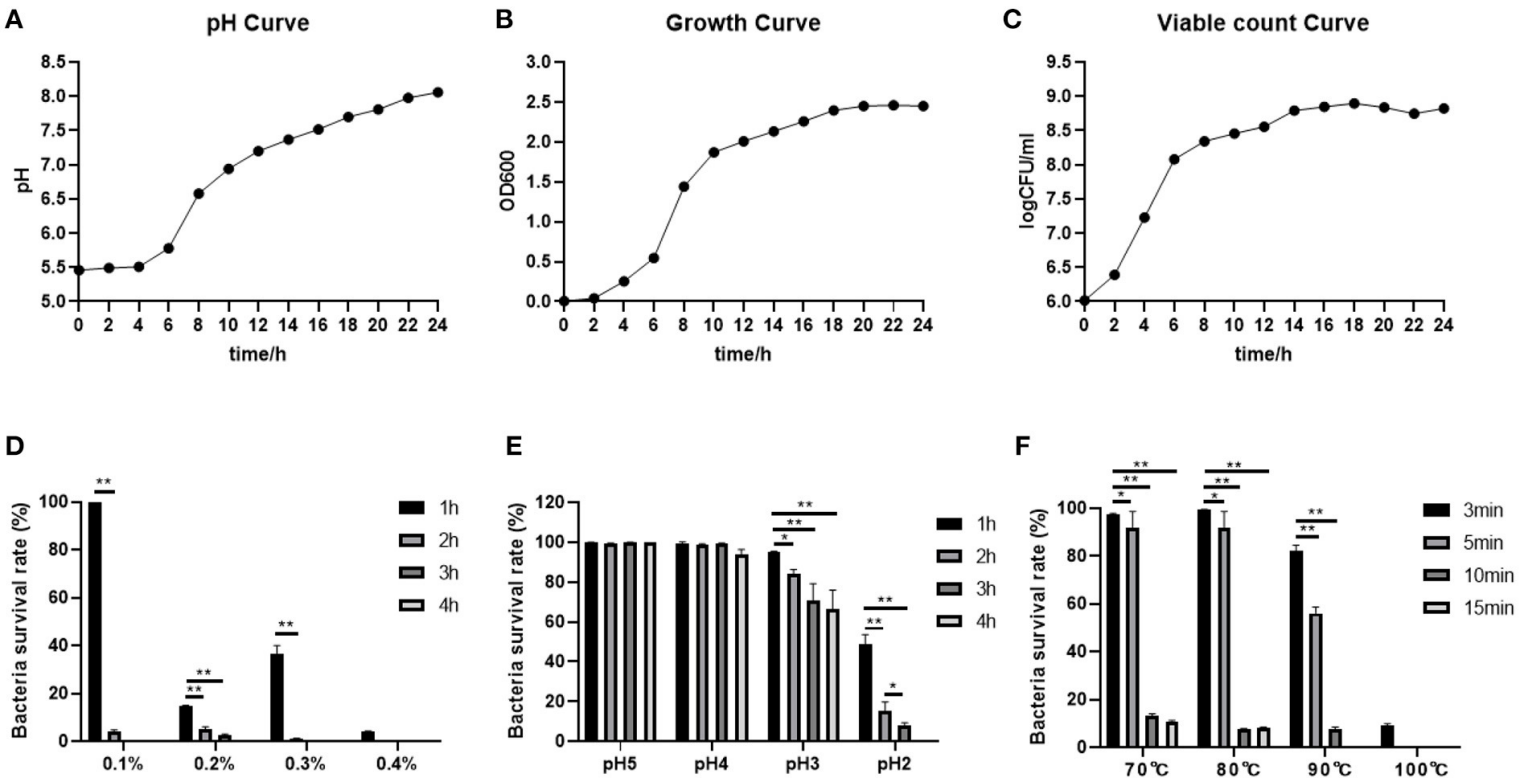
Probiotic properties of *B. subtilis M6*. **(A–C)** The pH curve, growth curve and viable count curve of *B. subtilis M6*. **(D)** The ability of bile salt tolerance of *B. subtilis M6*. **(E)** The ability of acid tolerance of *B. subtilis M6*. **(F)** The temperature sensitivity of *B. subtilis M6*. *n* = 6. *P < 0.05; **P < 0.01.

The absorbance values of the cultures at OD600 showed that the absorbance values increased rapidly from 2 to 18 h, indicating that the *B. subtilis M6* was in a logarithmic growth phase, and the absorbance gradually stabilized after 18 h and was in the plateau phase (Figure 2A). The number of viable bacteria increased rapidly from the 1 × 10^2^ CFU / ml to the 1 × 10^7^ CFU / ml at 2~14 h, after which the number of viable bacteria tended to be stable (Figure 2B). The pH increased rapidly in the first 14 h, and then the speed slowed down after 14 h. After 24 h of culture, the pH was 8.06, indicating that the strain has a strong alkali production ability (Figure 2C).

#### Bile salts, pH, and temperature tolerance of *B. subtilis M6*

The bile salt tolerance of the strain is relatively general. The addition amount of bile salt is 0.1%, and the survival rate is 100% after 1 h. After 2 h, it decreases sharply, and the survival rate is only 4% (Figure 2D). *B. subtilis M6* has strong acid resistance. It can survive normally under conditions of pH 4 and pH 5. At pH 3, with the decrease of the extension of culture time, its survival rate shows a downward trend. The survival rate of pH 2 for 2 h was higher than 10% (Figure 2E). As for high temperature, *B. subtilis M6* survival rate reaches 10.42% after withstanding for 15 min at 70°C, 8.03% after withstanding for 15 min at 80°C, 7.77% after withstanding for 10 min at 90°C, and 9.15% after withstanding for 3 min at 100°C (Figure 2F).

#### Antimicrobial activity of *B. subtilis M6*

We used the bacterial suspension, supernatant, and sediment of *B. subtilis M6* to test its bacteriostatic effect on common pathogenic *Escherichia coli, Salmonella*, and *Staphylococcus* (Table 1). The results showed that the bacterial suspension of *B. subtilis M6* had a bacteriostatic effect on all 10 pathogenic bacteria involved in the detection. *E. coli* K88 and *E. coli* K99 had a bacteriostatic effect, but the supernatant and precipitate of *B. subtilis M6* had no bacteriostatic effect on *E. coli* K88 or *E. coli* K99 (Supplementary Figure 1). *B. subtilis M6* had the best antibacterial effect on *Staphylococcus epidermidis*, and the diameter of the antibacterial circle reached 17 mm. For *Salmonella*, the inhibition zones of the three assays were all 12~15 mm.

**Table 1:** The diameter of the inhibition zone of *B. subtilis M6* against pathogenic bacteria.

**Indicator species**	**Inhibitory zone (mm)**		
	**Fermentation broth**	**Sediment**	**Suspension**
*E. coli* K88	13	-	-
*E. coli* K99	14	-	-
*S. aureus* CVCC1882	15	13	13
*S. aureus* ATCC25923	14	12	12
*S. aureus* ATCC43300	15	-	14
*S. aureus* ATCC6538	15	13	14
*S. pullorum* CVCC 519	15	-	14
*S. pullorum* ATCC14028	15	12	14
*S. typhimurium* CMCC50115	16	12	13
*S. epidermidis* ATCC12228	17	17	16

#### Antibiotic sensitivity of *B. subtilis M6*

Twenty-eight antibiotics from different broad classes were tested in this study (Table 2). *B. subtilis M6* is sensitive to 24 antibiotics such as Amikacin, Cefradine, Carbenicillin, Norfloxacin, and Gentamicin, etc. It also showed moderate sensitivity to Penicillin, Ceftazidime, Piperacillin, and Ampicillin. *B. subtilis M6* only showed drug resistance to Polyxin B (Supplementary Figure 2).

**Table 2:** Antibiotic sensitivity of *B. subtilis M6*.

**Antibiotic names**	**Content (ug/pill)**	**Inhibition zone (mm)**	**Sensitivity**
Amikacin (AK)	30	31	Sensitive
Cefradine (RAD)	30	58	Sensitive
Carbenicillin (CB)	100	26	Sensitive
Norfloxac (NOR)	10	39	Sensitive
Gentamicin (GM)	10	31	Sensitive
Tetracycline (TE)	30	23	Sensitive
Chloramphenicol (C)	30	38	Sensitive
Vancomycin (VA)	30	23	Sensitive
Erythromycin (E)	15	34	Sensitive
Cefoperazone (CFP)	75	34	Sensitive
Minocycline (MI)	30	39	Sensitive
Ceftriaxone (CTR)	30	29	Sensitive
Kanamycin (K)	30	32	Sensitive
Furazolidone (FZ)	300	31	Sensitive
Clindamycin (CC)	2	29	Sensitive
Ofloxacin (OFX)	5	39	Sensitive
Sulfamethoxazole (SXT)	23.75	35	Sensitive
Neomycin (N)	30	25	Sensitive
Midecamycin (MD)	30	30	Sensitive
Cefazolin (CZ)	30	52	Sensitive
Cefuroxime (CXM)	30	29	Sensitive
Ciprofloxacin (CIP)	5	41	Sensitive
Doxycycline (DX)	30	34	Sensitive
Penicillin (P)	10 U	17	Moderate sensitive
Ceftazidime (CAZ)	30	20	Moderate sensitive
Piperacillin (PIP)	100	20	Moderate sensitive
Ampicillin (AM)	10	20	Moderate sensitive
Polymyxin B (PB)	300 IU	10	Drug resistance

The circle diameter <15 mm was resistant and 16~20 mm for medium sensitive, the bacteriosphere diameter of >20 mm is sensitive.

### Identifiers effect of *Bacillus* in feed on growth performance of AA broilers

Promoting animal production performance and reducing feed conversion rate are considered one of the characteristics of probiotics. In this study, *B. subtilis M6* supplementation enhanced the performance of the experimental AA broilers. The body weight of AA broilers on the 21^st^ day, the body weight on the 42^nd^ day, and the average daily gain were significantly increased by adding *Bacillus* to the feed (Table 3). The addition of *Bacillus* had no significant effect on the spleen index, bursa index, and thymus index of AA broilers (Supplementary Table 1).

**Table 3:** Effects of different treatments on growth performance in AA broilers.

**Items**	**CON**	**CON+**	**TRT**	**SEM**	***P***
Initial weight (g)	47.83	48.15	47.04	0.24	0.893
Weight of 21 day (g)	385.31^a^	377.70^a^	403.28^b^	3.61	0.004
Weight of 42 day (g)	1271.08^a^	1273.885^a^	1338.255^b^	10.17	0.003
**Phase1 (1–21 d)**					
ADG (g/d)	16.07^a^	15.69^a^	16.96^b^	0.18	0.004
ADFI (g/d)	24.85	25.12	25.87	0.27	0.302
FCR (F/G)	1.55	1.60	1.52	0.02	0.125
**Phase 2 (22–42 d)**					
ADG (g/d)	42.18^a^	42.68 ^a^	44.52 ^b^	0.37	0.013
ADFI (g/d)	80.93	79.37	82.27	0.74	0.294
FCR (F/G)	1.92	1.86	1.85	0.02	0.425
**All phase (1–42 d)**					
ADG (g/d)	29.13 ^a^	29.18 ^a^	30.74 ^b^	0.24	0.002
ADFI (g/d)	52.90	52.25	54.07	0.38	0.143
FCR (F/G)	1.82	1.79	1.76	0.01	0.299

CON, basic diet; CON+, basic diet + 200 ppm aureomycin; TRT, basic diet + 1 × 10^9^CFU / g B. subtilis M6. ADG: average daily gain; ADFI: average daily feed intake; FCR: feed conversion ratio; SEM: standard error of the mean; Different letter superscripts in the same row indicate a significant difference (P < 0.05). n = 6.

### Effect of *Bacillus* in feed on intestinal morphology of AA broilers

**Figure 3:**
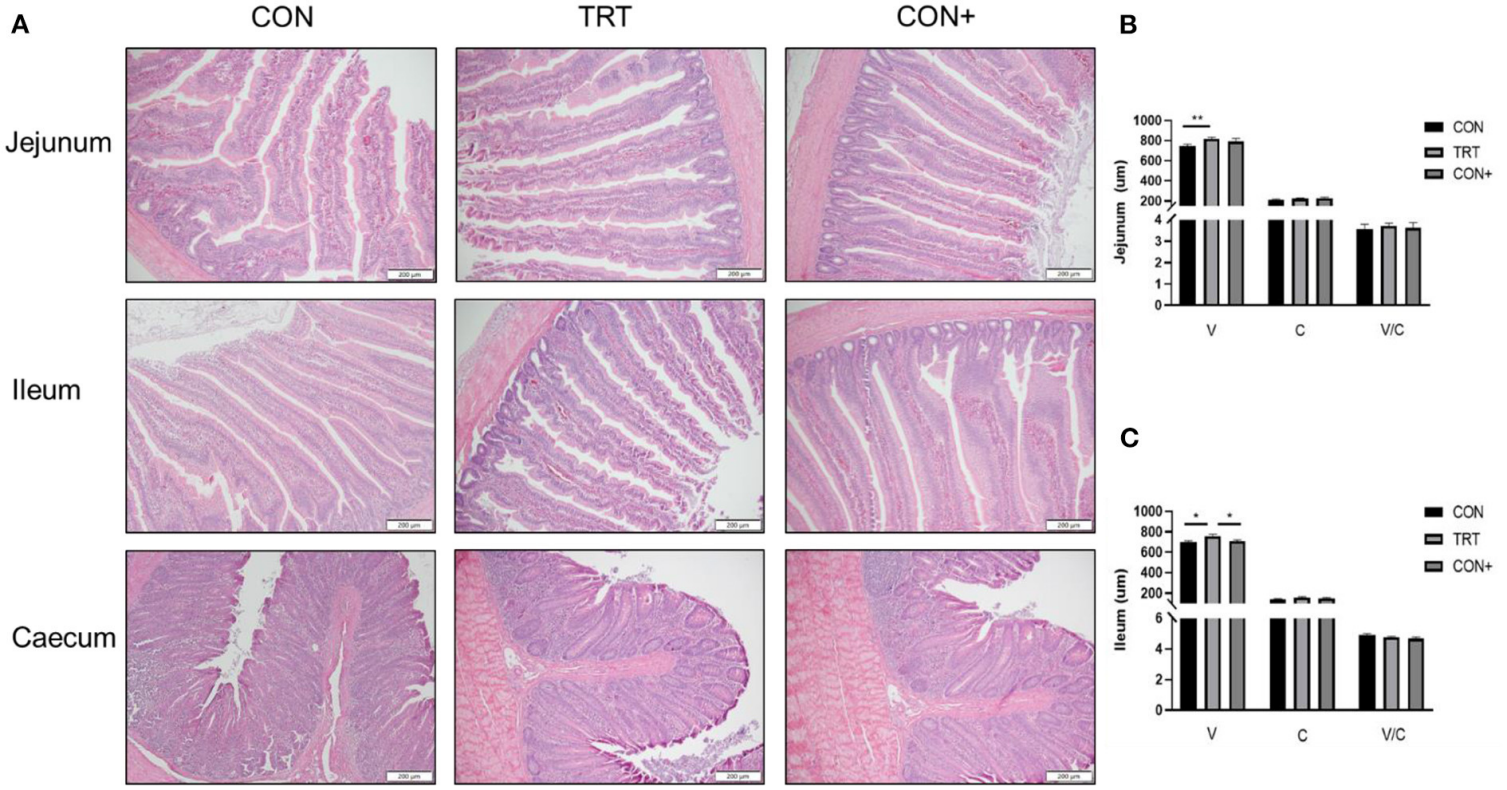
Effect of different treatments on intestinal morphology. **(A)** Intestinal morphology of the jejunum, ileum and caecum in AA broilers. Representative pictures of different intestinal segments were shown, bar = 200 μm. **(B)** Height of jejunum villi in different treatment groups. **(C)** Height of ileum villi in different treatment groups. *n* = 6. *P < 0.05; **P < 0.01.

The tissue structure of each intestinal segment of AA broilers in the TRT group was clear and small intestinal villi were tightly arranged (Figure 3A). The results showed that when compared to the control group, the addition of *Bacillus* in the feed could significantly increase the height of the jejunal villus but had no significant effect on the depth of the jejunal recess and the jejunal villus ratio (Figure 3B). For the ileum, the addition of *Bacillus* in the feed could significantly increase the height of the ileal villus but had no significant effect on the height of the ileal villus and the ratio of the ileal villus (Figure 3C).

### Effects of *B. subtilis M6* supplementation on the intestinal microflora of broilers

#### Effects of *B. subtilis M6* on microbial composition of the caecum

**Figure 4:**
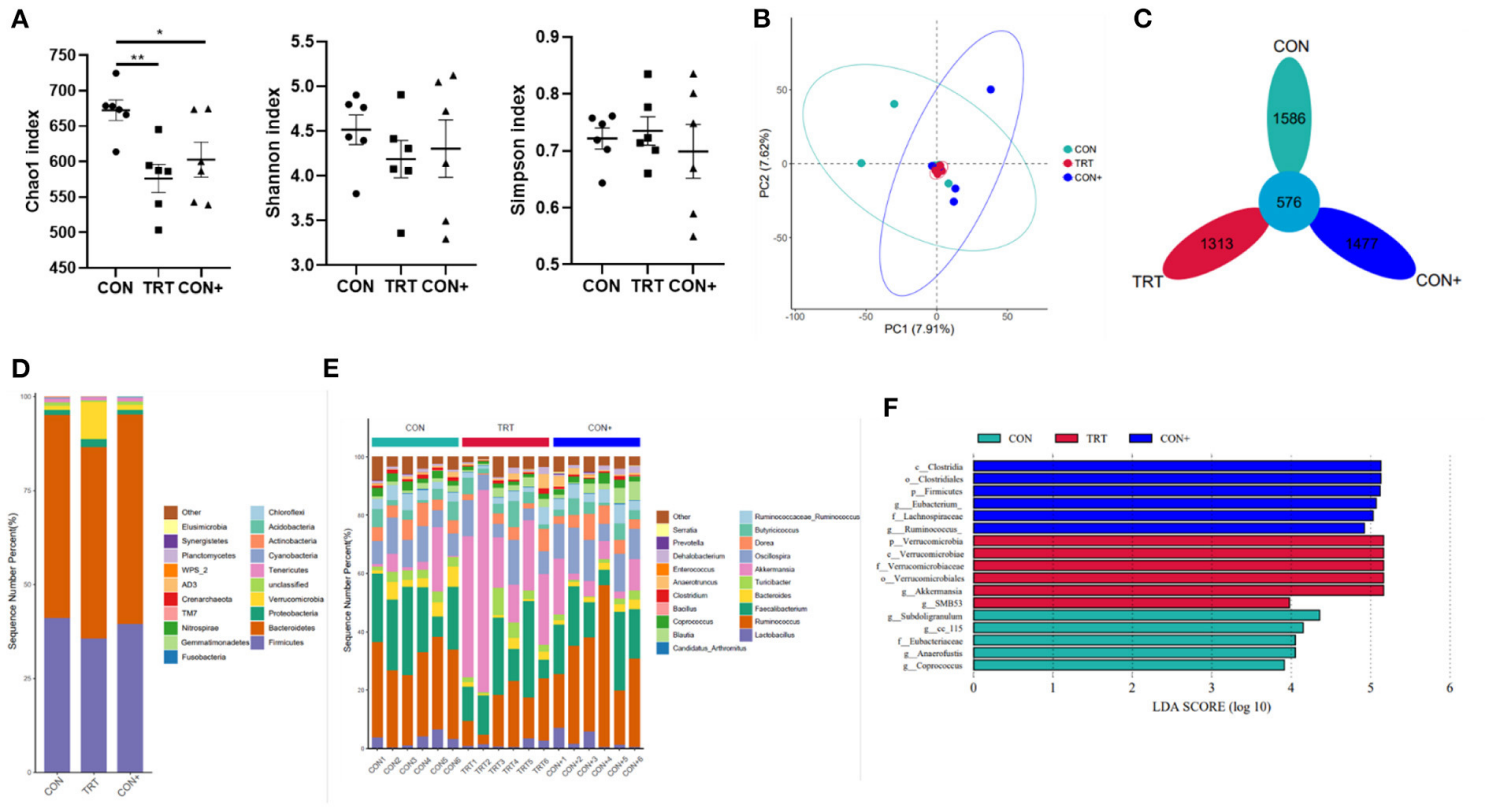
Effects of *B. subtilis M6* microbial composition of caecum. **(A)** The α-diversity comparisons was analyzed by Chao1, Shannon's diversity and Simpson index, data was shown as mean ± SEM. **(B)** The β-diversity comparisons was analyzed by PCA. **(C)** Common species analysis was shown by Petal diagram. **(D)** Community composition of the gut microbiota at the phylum levels. **(E)** Community composition of the gut microbiota at the genus levels. **(F)** Bacterial taxa differentially was identified by LEFSe using an LDA score threshold of >2.0 and *P* < 0.05. *n* = 6. *P < 0.05; **P < 0.01.

To evaluate the effect of the *B. subtilis M6* on the intestinal microbial composition of AA broilers, the caecum chyme samples were analyzed by 16S rDNA sequencing. Compared to the Con group, the Shannon and Simpson indexes in the TRT group significantly changed but the Chao index significantly decreased (Figure 4A). According to β diversity, Principal Component Analysis (PCA) showed that caecum community composition of the CON group, CON+ group and TRT group was not significantly discriminated from each other (Figure 4B). According to the common strains analyzed by the Flower diagram, there were 576 common strains between three groups (Figure 4C).

At the phylum level, the most abundant phyla in the caecum with three groups were Bacteroidetes, followed by Firmicutes. Compared with control group, the TRT group showed a decrease in the abundance of the Firmicutes (41.05 and 35.62%, respectively). In addition, the abundance of Verrucomicrobia (9.79%), and Proteobacteria (2.16%) in the TRT group were higher than in the control group (1.16% and 1.21% in the control group, respectively) (Figure 4D, Supplementary Table 2). The abundance of different phyla in the CON group compared with the CON+ group was generally consistent.

The genus level was further analyzed, and the results showed that above 80% was unclassified (Supplementary Table 2). The results of the analysis of the classified genera are presented in Figure 4E. The dominant genus in the control group was *Ruminococcus* (5.66%), but in the TRT group, the dominant genus was changed to *Akkermansia* (9.79%) but not *Ruminococcus* (3.32%). In addition, the treatment TRT group increased the abundance of *Faecalibacterium* (4.92%), *Oscillospira* (1.96%), and *Butyricicoccus* (1.00%) than the CON group (4.08, 1.72 and 0.78% in CON group, respectively). The abundance of *Dorea* (0.67%) and *Ruminococcus* (0.60%) were decreased in the TRT group as compared with the CON group (0.97 and 0.74% in the CON group respectively).

LEfSe analysis of the taxa showed that 15 OTUs were differentially present between three groups in the caecum (Figure 4F). It shows more visually the characteristic dominant bacteria in the different treatment groups.

#### Effects of *B. subtilis M6* on microbial composition of the ileum

The ileum microbial composition analysis was further explored in Supplementary Figure 3, the result showed that compared with the Con group, the Shannon and Simpson indexes in the TRT group and CON+ group were significantly increased (Supplementary Figure 3A). The Chao index was also higher than the CON group (*P* > 0.05). According to β diversity, principal coordinates analysis (PCoA) based on the weighted normalized unifrac method showed that the samples in the CON+ group were more dispersed. The samples in the TRT group were clearly separated from the CON and CON+ groups (Supplementary Figure 3B). The flower diagram showed that there are 198 common strains between three groups in the ileum, and 2,361 differential strains in the TRT group (Supplementary Figure 3C).

At the phylum level, the most abundant phylla in the ileum of the CON group, TRT group, and CON+ group are Firmicutes (94.89, 90.37, and 80.51%, respectively), followed by Proteobacteria (2.72, 3.84, and 14.28%). In addition, the abundance of Bacteroidetes (2.85%), Actinobacteria (0.38%), and Verrucomicrobia (0.49%) in the TRT group were higher than CON group (1.77%, 0.09%, and 0.08% in CON group respectively) (Supplementary Figure 3D, Supplementary Table 4).

The genus level was further analyzed, and the results showed that in the CON group and CON+ group above 80% were unclassified (89.06 and 77.94%respectively). The dominant genus in the TRT group was *Lactobacillus* (70.28%) followed by *Turicibacter* (1.85%). It was decreased than the CON group (6.67%) and CON+ group (4.49%). In addition, the treatment of TRT group increased the abundant of *Candidatus_Arthromitus* (1.46%), *Ruminococcus* (0.59%), *Bacillus* (0.87%), *Enterococcus* (0.63%) and *Bacteroides* (0.59%) than control group (0.22%, 0.38%, 0.05%, 0.06% and 0.03% in the control group respectively). To compare with the CON+ group, the treatment of the TRT group increased the abundance of *Candidatus_Arthromitus* (1.46%) (0.53% in the CON+ respectively) (Supplementary Figure 3E, Supplementary Table 5).

LEfSe analysis of the taxa showed that 34 OTUs were differentially present between three groups in the ileum (Supplementary Figure 3F). It shows more visually the characteristic dominant bacteria in the different treatment groups.

### Effects of *B. subtilis M6* on intestinal antioxidant function of AA broilers

The indexes related to antioxidation in jejunum and ileum mucosa of AA broilers were tested, and the results are shown in the Table 4. For jejunal mucosa, the content of malondialdehyde (MDA) in the CON+ group was significantly lower than that in the CON group and TRT group, while the contents of superoxide dismutase (SOD) and total antioxidant capacity (T-AOC) had no significant difference between those three groups. For ileal mucosa, the content of MDA in the TRT group was significantly lower than that in the CON group and CON+ group, but there was no significant difference SOD and T-AOC (Table 4).

**Table 4:** Antioxidant effect of jejunum and ileum in AA broilers.

**Items**	**CON**	**CON+**	**TRT**	**SEM**	***P***
**Jejunum**
T-SOD (U/mgprot)	9.87	7.34	8.61	0.6267	0.2868
T-AOC (U/mgprot)	2.36	1.72	2.31	0.1463	0.1205
MDA (nmol/mgprot)	1.75^a^	1.38^b^	1.81^a^	0.0674	0.0054
**Ileum**
T-SOD (U/mgprot)	11.12	7.81	8.81	1.0326	0.4437
T-AOC (U/mgprot)	1.75	2.27	1.55	0.2103	0.3736
MDA (nmol/mgprot)	2.25^a^	1.65^a^	1.54^b^	0.1307	0.0275

CON, basic diet; CON+, basic diet + 200 ppm aureomycin; TRT, basic diet + 1 × 10^9^CFU / g B. subtilis M6. SEM means standard error of the mean, different letter superscripts in the same row indicate a significant difference (P < 0.05), n = 6.

## Discussion

Many antibiotics are used as feed additives in the Chinese farming industry to promote growth performance. However, the heavy use of antibiotics may lead to dysbiosis, resistance, and even antibiotic-associated diarrhea. Therefore, the ban on antibiotics in feed has been enforced and it is urgent to find an additive that can replace the role of antibiotics. There is growing evidence that supplementation with probiotics can promote animal growth and inhibit the colonization of pathogenic microorganisms (10). *Bacillus* can produce a variety of enzymes (such as protease, amylase, and lipase), antibacterial metabolites, and biopeptides (11). The metabolites produced by this bacillus can help to improve the digestion and absorption of nutrients in the animal intestine, inhibit the growth of pathogenic bacteria and regulate the intestinal microbiota. *Bacillus* is widely used in dietary supplements and therapeutic drugs in humans, as a growth promoter and competitive exclusion agent in animals, and in aquaculture to enhance the growth and disease resistance of shrimp (11).

Before performing the animal experiment, some *in vitro* tests were performed to evaluate the antibacterial activity and safety of *B. subtilis M6*, including growth characteristics, acid, and bile salts tolerance tests, temperature tolerance tests, antibacterial tests, and antibiotic susceptibility assay. *Bacillus* can produce bacteriocin and has a wide range of antibacterial activities, so they are used as antifungal agents (12), antiviral agents (13), and anti-mycoplasma agents (14). And the spores of *Bacillus* can survive at low pH, which is in line with the needs of the animal stomach environment (15). The use of antibiotics will cause the drug-resistant gene to spread to other microorganisms in the digestive tract, which will pollute the environment and spread to other bacteria in the environment. The serious problem of drug resistance will affect human beings, resulting in the unavailability of antibiotics. Of the 28 antibiotics involved in the test, *B. subtilis M6* showed resistance to only one antibiotic, indicating that the use of this strain does not pose a safety concern. *M6* exhibits excellent antibacterial properties and can inhibit the multiplication of pathogenic *E. coli, Salmonella* and *Staphylococcus*. The results showed that *M6* had the potential as probiotics.

In humans and animals, major nutrient absorption occurs in the small intestine and is positively correlated with the length of the small intestinal villi (16). Previous studies have shown that probiotic supplementation increases the length of the intestinal villi and decreases their crypt depth (17). In this study, a significant increase in the height of intestinal villi in both jejunum and ileum was found in the *B. subtilis M6* treated group compared to the control group, which is consistent with the results of previous studies. It is known that the growth and development of animals cannot separate from the absorption of nutrients. Studies have shown that *Bacillus* is a probiotic, which is widely used to prevent gastrointestinal diseases and improve animal growth performance (18–20). Under the condition of oxidative damage, bacillus can effectively improve the intestinal morphology and integrity of rats (21). Our study just showed that *B. subtilis M6* can significantly improve the ADG and BW of AA broilers, which is consistent with previous studies. AA broilers supplemented with *B. subtilis M6* have higher final body weight without increasing food intake, which means that the probiotic-treated broilers possess better absorption and digestion capabilities. In the study of calves, it was also confirmed that feeding *Bacillus* can promote the weight gain of calves and show therapeutic potential in growth performance by regulating hormones and improving the development of intestinal and rumen with growth retardation (22). Our results were consistent with previous studies that probiotics can promote animal growth by improving small intestinal mucosa morphology.

Probiotic strains, such as *Bifidobacterium, Lactobacillus*, and *Bacillus*, have been shown to have a strong antioxidant capacity and reduce oxidative damage *in vivo* and *in vitro* (23–25). And some recent studies have shown that some *Bacillus* strains are beneficial to prevent oxidative stress (26, 27). Free radical ROS can cause membrane lipid peroxidation. The concentration of MDA increased significantly under oxidative damage, while the concentration of MDA decreased significantly under the treatment of probiotics (21). In our study, the concentration of MDA in ileal mucosa was detected. It was found that *B. subtilis M6* can significantly reduce the concentration of MDA, which is consistent with the previous research results.

Intestinal microbiota plays an important role in digesting food and absorbing nutrients from the host diet, regulating host fat storage, stimulating intestinal epithelial renewal, and guiding the maturation of the immune system (28). The use of antibiotics in animal husbandry breeding not only improves the growth performance but also introduces problems (29). Whether the therapeutic or preventive use of antibiotics will disturb the normal microbiota balance of the host, which may lead to the growth retardation of animals (30). Probiotics are used as a safe alternative to antibiotics to rebalance the intestinal flora and supplement the diet to prevent disease, improve inflammation and digestion and promote growth (31, 32). These mechanisms of beneficial effects on the host have been verified and involve interference with potential pathogens, improvement of barrier function, immune regulation, and production of neurotransmitters (33). At present, lactic acid bacteria and *Bacillus subtilis* are used as feed additives to improve the growth performance and immune function of some animals (34).

Previous studies have reported that the diversity, composition, and relative abundance of intestinal flora are affected by probiotic administration. Feeding probiotics to animals can regulate microbial diversity (35, 36). Symbiotic microorganisms play a role in many aspects of animal biology, including digestion and absorption, immune development, behavior, and development. It is reported that Feeding Broilers with *Bacillus* cereus fermented feed optimizes the intestinal flora of Broilers and further improves the digestion and absorption capacity of broilers (37). We observed a significant increase in *Akkermansia* in cecum chyme in the TRT group. Previous studies found that *Akkermansia muciniphila* secretes a glucagon-like peptide-1-inducing protein that improves glucose homeostasis in mice (38). In addition, *Akkermansia* has also been found to have a good effect on regulating host intestinal health and improving immunity (39). The growth performance of broilers is closely related to the diversity of cecal flora fermented by chime (40). From this, we can infer that the addition of *B. subtilis M6* has a positive effect on the development of microbial communities in the cecum. Probiotics can inhibit the growth of pathogenic bacteria and change the overall structure of the cecal microbial community, which is conducive to the intestinal health of broilers (41). This is consistent with our research results.

In summary, our results showed that *B. subtilis M6* supplementation increased intestinal antioxidant capacity and regulated the microbial community, especially at the level of the caecum genus, the dominant genus was changed from *Ruminococcus* to *Akkermansia*. Our study suggests that *B. subtilis M6* supplementation improves the growth performance of AA broilers, and the potential mechanisms may be attributed to the effects on gut microbial composition and absorption of nutrients by the intestine. In addition, our studies also support the increasing scientific evidence that probiotic supplementation may have a healthy and beneficial effect on the host.

## Data availability statement

The data presented in the study are deposited in the NCBI Sequence Read Archive (SRA) repository, accession number PRJNA847429.

## Ethics statement

The animal study was reviewed and approved by Animal Protection and Utilization Organization Committee of China Agricultural University. Written informed consent was obtained from the owners for the participation of their animals in this study.

## Author contributions

XM and XZ conceived and designed the experiments. LJ, LZ, and YZ conducted most of the animal experiments and contributed to collecting samples. LZ, LJ, and JS performed all of the *in vitro* bacteria experiments. LJ performed the statistical analysis for all the data and wrote the original draft. HL, XZ, and XM gave a critical reading and editing. XM resourced the project. All authors read and approved the final manuscript.

## Funding

This work was supported by the National Natural Science Foundation of China (31930106 and 31829004), the Henan Province Public Benefit Research Foundation (201300111200-05), the 2115 Talent Development Program of China Agricultural University (1041-00109019), and the 111 Project (B16044).

## Conflict of interest

The authors declare that the research was conducted in the absence of any commercial or financial relationships that could be construed as a potential conflict of interest.

## Publisher's note

All claims expressed in this article are solely those of the authors and do not necessarily represent those of their affiliated organizations, or those of the publisher, the editors and the reviewers. Any product that may be evaluated in this article, or claim that may be made by its manufacturer, is not guaranteed or endorsed by the publisher.
