# Supplementary material for: Bacillus subtilis M6 improves intestinal barrier, antioxidant capacity and gut microbial composition in AA broiler
Source: Front Nutr. 2022 Aug 17;9:965310. doi: 10.3389/fnut.2022.965310 (PMC9428444; doi:10.3389/fnut.2022.965310)
Supplement: Supplementary file 1 [file Data_Sheet_1.docx]

**Supplementary materials**

**Supplementary Figure 1.** Antibacterial activity of *B. subtilis* M6. The inhibitory effect of *B. subtilis* M6 against common pathogenic bacteria. Hole 1 is the fermentation broth of the *B. subtilis* M6. Hole 2 is the sediment of *B. subtilis* M6. Hole 3 is the group of bacterial suspensions of *B. subtilis* M6. The middle is a negative control.


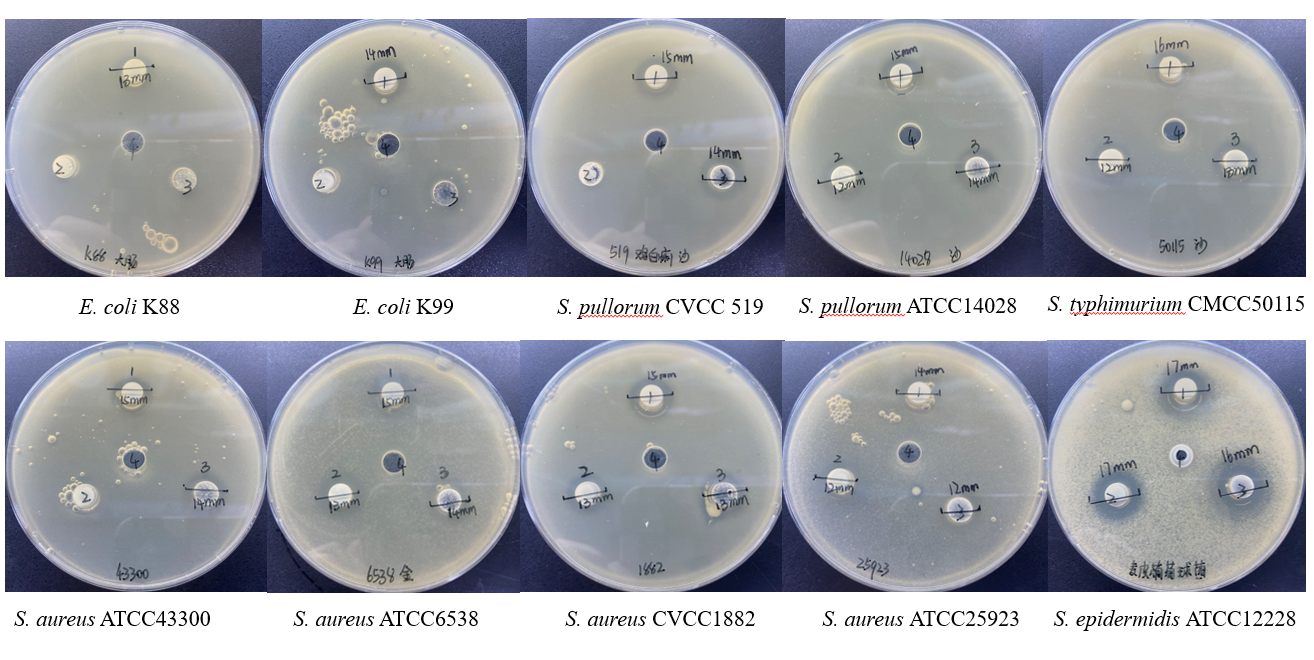


**Supplementary Figure 2.** Antibiotic sensitivity of *B. subtilis* M6.


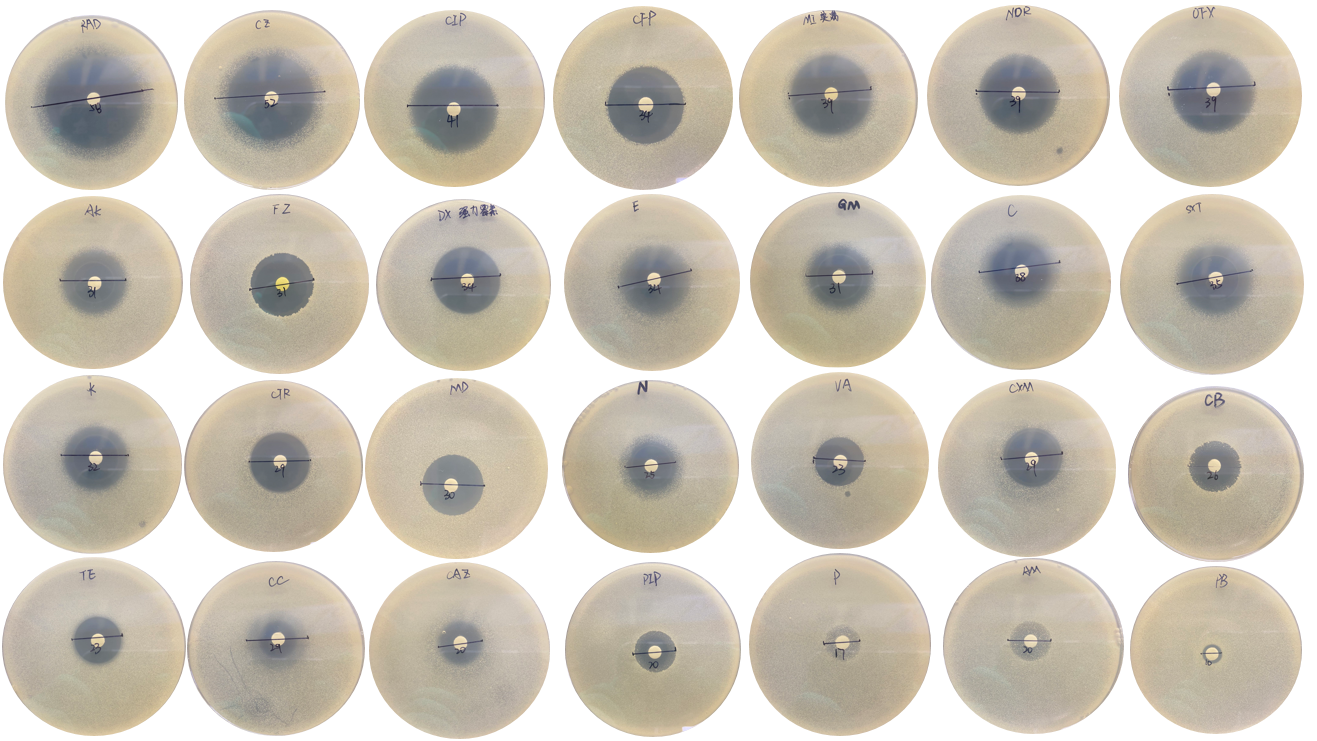


**Supplementary Figure 3.** Effects of *B. subtilis* M6 microbial composition of the ileum

(A) The α-diversity comparisons was analyzed by Chao1 index, Shannon index and Simpson index, data were shown as mean ± SEM. (B) The β-diversity comparisons were analyzed by weighted UniFrac PCoA. (C) Common species analysis was shown by the Flower diagram. (D) Community composition of the gut microbiota at the phylum levels. (E) Community composition of the gut microbiota at the genus levels. (F) Bacterial taxa differentially were identified by LEFSe using an LDA score threshold of >2.0 and *P*<0.05.


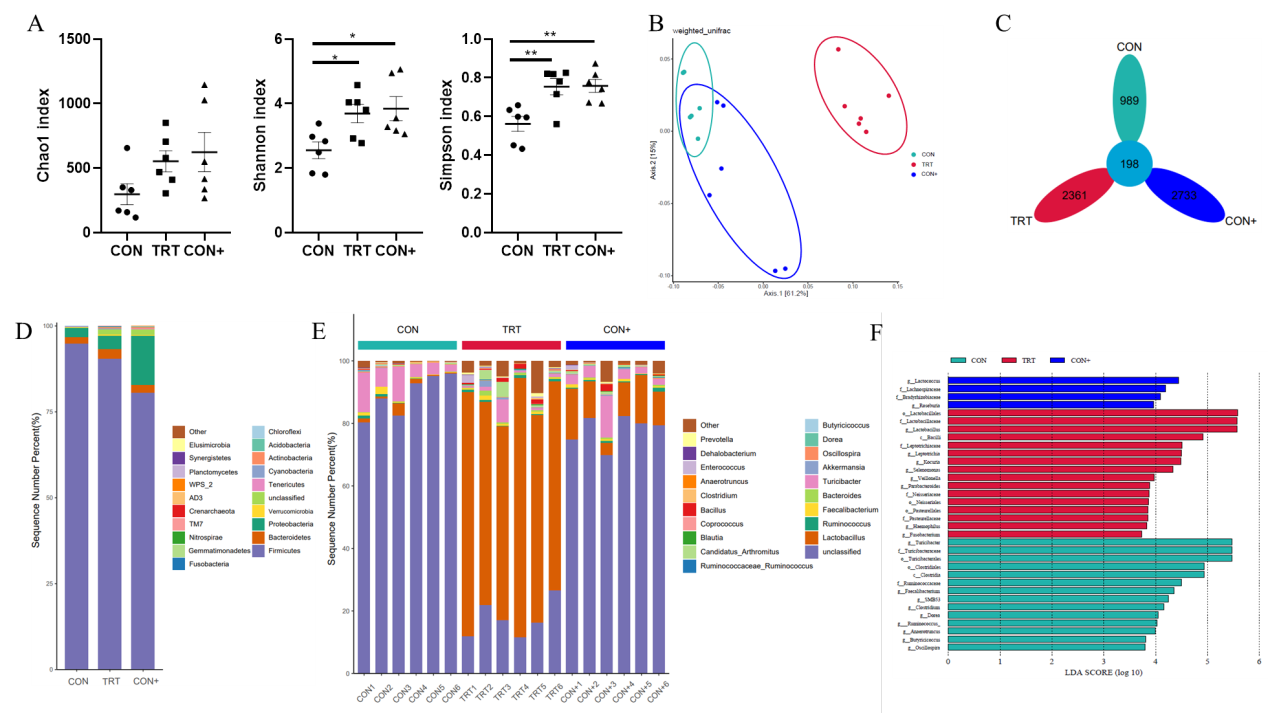


**Supplementary Table S1**. Organ index of AA broiler

| Items | CON | CON+ | TRT | SEM | *P* |
| --- | --- | --- | --- | --- | --- |
| Spleen index | 1.09 | 1.40 | 1.45 | 0.068 | 0.521 |
| Bursa of fabricius index | 2.15 | 2.06 | 2.31 | 0.141 | 0.793 |
| Thymus index | 1.61 | 1.98 | 1.86 | 0.097 | 0.290 |

Note: CON, basic diet; CON+, basic diet + 200ppm aureomycin; TRT, basic diet + 1 × 10^9^CFU / g *B. subtilis* M6. SEM means standard error of the mean, n = 12.

**Supplementary Table S2** Community composition of the gut microbiota in caecum at the phylum level

|  | CON | TRT | CON+ |
| --- | --- | --- | --- |
| Bacteroidetes | 0.540992191 | 0.509363 | 0.558122 |
| Firmicutes | 0.410509809 | 0.356233 | 0.394411 |
| Verrucomicrobia | 0.011643584 | 0.097919 | 0.013669 |
| Proteobacteria | 0.012119051 | 0.02163 | 0.011875 |
| Tenericutes | 0.009491102 | 0.008969 | 0.009781 |
| unclassified | 0.008739483 | 0.004153 | 0.008152 |
| Cyanobacteria | 0.004607107 | 0.001192 | 0.003126 |
| Actinobacteria | 0.001685251 | 0.000471 | 0.000652 |
| Acidobacteria | 0.000111734 | 1.77E-05 | 0.000106 |
| Euryarchaeota | 3.18E-05 | 2.03E-05 | 1.63E-05 |
| Gemmatimonadetes | 1.49E-05 | 0 | 4.34E-05 |
| Chloroflexi | 1.88E-05 | 0 | 2.50E-05 |
| Lentisphaerae | 1.19E-05 | 1.16E-05 | 0 |
| GN02 | 1.73E-05 | 0 | 0 |
| Fusobacteria | 6.05E-06 | 0 | 6.53E-06 |
| OD1 | 0 | 8.55E-06 | 0 |
| Thermi | 0 | 0 | 7.98E-06 |
| Crenarchaeota | 0 | 0 | 6.21E-06 |
| AD3 | 0 | 5.70E-06 | 0 |
| Chlamydiae | 0 | 5.70E-06 | 0 |

**Supplementary Table S3** Community composition of the gut microbiota in caecum at the genus level

|  | CON | TRT | CON+ |
| --- | --- | --- | --- |
| *unclassified* | 0.807085 | 0.73711 | 0.808156 |
| *Ruminococcus* | 0.05657 | 0.033168 | 0.061132 |
| *Akkermansia* | 0.011638 | 0.097913 | 0.013653 |
| *Faecalibacterium* | 0.04079 | 0.049162 | 0.031062 |
| *Oscillospira* | 0.017207 | 0.019581 | 0.02355 |
| *Dorea* | 0.009653 | 0.006666 | 0.01024 |
| *Butyricicoccus* | 0.00777 | 0.010049 | 0.007232 |
| *Ruminococcus* | 0.007369 | 0.005976 | 0.008151 |
| *Turicibacter* | 0.005691 | 0.008538 | 0.00267 |
| *Lactobacillus* | 0.005923 | 0.004324 | 0.005662 |
| *Bacteroides* | 0.007908 | 0.003679 | 0.003837 |
| *Blautia* | 0.002722 | 0.004789 | 0.00555 |
| *Coprococcus* | 0.004767 | 0.003348 | 0.004174 |
| *Anaerotruncus* | 0.001458 | 0.002763 | 0.002781 |
| *Dehalobacterium* | 0.001618 | 0.002045 | 0.002413 |
| *Clostridium* | 0.001983 | 0.00159 | 0.001199 |
| *Anaeroplasma* | 0.001951 | 0.000978 | 0.001822 |
| *rc4_4* | 0.000499 | 0.001141 | 0.000944 |
| *SMB53* | 0.000489 | 0.00164 | 0.000122 |
| *cc_115* | 0.000778 | 0.000195 | 0.000648 |
| *Other* | 0.006131 | 0.005347 | 0.005003 |

**Supplementary Table S4** Community composition of the gut microbiota in ileum at the phylum level

|  | CON | TRT | CON+ |
| --- | --- | --- | --- |
| Firmicutes | 0.948959699 | 0.90375 | 0.805135 |
| Proteobacteria | 0.02726707 | 0.038426 | 0.142842 |
| Bacteroidetes | 0.017748991 | 0.028534 | 0.023601 |
| unclassified | 0.002623387 | 0.013425 | 0.016894 |
| Actinobacteria | 0.000920954 | 0.00383 | 0.002873 |
| Verrucomicrobia | 0.000787117 | 0.004944 | 0.001494 |
| Cyanobacteria | 0.000302197 | 0.002675 | 0.001178 |
| Acidobacteria | 0.000358773 | 0.000957 | 0.002309 |
| Tenericutes | 0.000616445 | 0.001201 | 0.001702 |
| Chloroflexi | 0.000166254 | 0.00062 | 0.000533 |
| Fusobacteria | 2.48E-05 | 0.000891 | 0.000224 |
| Gemmatimonadetes | 4.14E-05 | 0.000162 | 0.000236 |
| Nitrospirae | 4.02E-05 | 5.50E-05 | 0.000237 |
| TM7 | 0 | 0.000164 | 4.48E-05 |
| WPS_2 | 0 | 1.27E-05 | 0.000173 |
| Crenarchaeota | 1.38E-05 | 7.42E-05 | 8.37E-05 |
| AD3 | 0 | 4.90E-05 | 8.99E-05 |
| Planctomycetes | 0 | 5.17E-05 | 5.75E-05 |
| Synergistetes | 6.31E-05 | 2.01E-05 | 1.56E-05 |
| Elusimicrobia | 0 | 4.97E-05 | 4.18E-05 |
| Other | 6.58E-05 | 0.00011 | 0.000236 |

**Table S5** Community composition of the gut microbiota in ileum at the genus level

|  | | CON | TRT | CON+ |
| --- | --- | --- | --- | --- |
| *unclassified* | 0.890641 | | 0.174668 | 0.779391 |
| *Lactobacillus* | 0.012809 | | 0.702862 | 0.11557 |
| *Turicibacter* | 0.066667 | | 0.018544 | 0.04494 |
| *Candidatus_Arthromitus* | 0.002237 | | 0.014573 | 0.005273 |
| *Faecalibacterium* | 0.007121 | | 0.00629 | 0.005705 |
| *Ruminococcus* | 0.003858 | | 0.005906 | 0.005374 |
| *Bacillus* | 0.000569 | | 0.008718 | 0.004818 |
| *Enterococcus* | 0.000627 | | 0.006328 | 0.003529 |
| *Bacteroides* | 0.000286 | | 0.005909 | 0.002469 |
| *Akkermansia* | 0.000762 | | 0.00485 | 0.001173 |
| *Serratia* | 0.000363 | | 0.003744 | 0.002258 |
| *Prevotella* | 0.000162 | | 0.004846 | 0.001037 |
| *Brevundimonas* | 8.52E-05 | | 0.004736 | 0.000689 |
| *Oscillospira* | 0.001503 | | 0.001443 | 0.001733 |
| *Weissella* | 3.10E-05 | | 0.004142 | 0.000143 |
| *Clostridium* | 0.001674 | | 0.001086 | 0.00075 |
| *SMB53* | 0.002011 | | 3.72E-05 | 0.000995 |
| *Streptococcus* | 0.000125 | | 0.001966 | 0.000513 |
| *Delftia* | 0.000105 | | 0.001806 | 0.000475 |
| *Dorea* | 0.000478 | | 0.001141 | 0.000682 |
| *Other* | 0.007883 | | 0.026406 | 0.022484 |
